# Single-Cell RNA Sequencing Reveals the Expansion of Cytotoxic CD4^+^ T Lymphocytes and a Landscape of Immune Cells in Primary Sjögren’s Syndrome

**DOI:** 10.3389/fimmu.2020.594658

**Published:** 2021-02-02

**Authors:** Xiaoping Hong, Shuhui Meng, Donge Tang, Tingting Wang, Liping Ding, Haiyan Yu, Heng Li, Dongzhou Liu, Yong Dai, Min Yang

**Affiliations:** ^1^ Department of Rheumatology and Immunology, Southern Medical University, Nanfang Hospital, Guangzhou, China; ^2^ Department of Rheumatology and Immunology, Department of Clinical Medical Research Center, Guangdong Provincial Engineering Research Center of Autoimmune Disease Precision Medicine, Shenzhen People’s Hospital (The Second Clinical Medical College of Jinan University, The First Affiliated Hospital Southern University of Science and Technology), Shenzhen, China

**Keywords:** primary Sjögren’s syndrome, single-cell RNA sequencing, CD4^+^ cytotoxic T lymphocytes, TCL1A, susceptibility genes

## Abstract

**Objective:**

Primary Sjögren’s syndrome (pSS) is a systemic autoimmune disease, and its pathogenetic mechanism is far from being understood. In this study, we aimed to explore the cellular and molecular mechanisms that lead to pathogenesis of this disease.

**Methods:**

We applied single-cell RNA sequencing (scRNA-seq) to 57,288 peripheral blood mononuclear cells (PBMCs) from five patients with pSS and five healthy controls. The immune cell subsets and susceptibility genes involved in the pathogenesis of pSS were analyzed. Flow cytometry was preformed to verify the result of scRNA-seq.

**Results:**

We identified two subpopulations significantly expand in pSS patients. The one highly expressing cytotoxicity genes is named as CD4^+^ CTLs cytotoxic T lymphocyte, and another highly expressing T cell receptor (TCR) variable gene is named as CD4^+^ TRAV13-2+ T cell. Flow cytometry results showed the percentages of CD4^+^ CTLs, which were profiled with CD4^+^ and GZMB^+^ staining; the total T cells of 10 patients with pSS were significantly higher than those of 10 healthy controls (*P*= 0.008). The expression level of IL-1β in macrophages, TCL1A in B cells, as well as interferon (IFN) response genes in most cell subsets was upregulated in the patients with pSS. Susceptibility genes including HLA-DRB5, CTLA4, and AQP3 were highly expressed in patients with pSS.

**Conclusions:**

Our data revealed disease-specific immune cell subsets and provided some potential new targets of pSS. Specific expansion of CD4^+^ CTLs may be involved in the pathogenesis of pSS, which might give valuable insights for therapeutic interventions of pSS.

## Introduction

Primary Sjögren’s syndrome (pSS) is one of the most common autoimmune diseases that mainly affect middle-aged and older women. Patients with pSS are characterized by extensive lymphocytic infiltration of the exocrine glands, especially the salivary glands and lacrimal glands, leading to oral and ocular dryness (1). Approximately 30–40% of patients will develop systemic complications involving the kidneys, lungs, nervous system, and other systems (2–4). Although current advances have increased the understanding of the disease complexity, including the recognition of disease heterogeneity, selection of candidate genes, and the elucidation of disease-related pathways (5, 6), the pathogenetic mechanism of pSS is far from being understood.

B cell hyperactivation plays a central role in the pathogenesis of pSS. At present, B cell activation mechanisms have made some progress, but the specific molecular mechanisms remain unclear (7, 8). The key role of B cells is inseparable from the involvement of T cells. The cellular characteristics have indicated that the infiltrating cells of salivary glands and lacrimal glands are dominated by CD4 T cells and B cells. In the early stages of the disease, the infiltrating cells are mainly CD4^+^ T cells (9). Activated T cells induce the activation of B cells by producing pro-inflammatory cytokines, establishing a positive feedback loop and contributing to the disease pathogenesis. Previous studies have evaluated the role of identified CD4 T cell subsets in pSS. For example, T follicular helper cells (T_FH_) contributed to the maturation of B cells by secreting IL-21 (Interleukin 21), T_H_17 cells may be expanded in patients with pSS and assisted autoreactive B cell response, promoting the disease progression (10, 11). Thus, defining the major cell subsets and their states is critical to explore new approaches to pSS therapy. Some specific cell subsets associated with pSS have been studied using flow cytometry or immunohistochemistry (IHC) analysis in salivary glands and PBMCs (12, 13). However, these studies were based on preselected cell types, and recent advances in high-resolution single-cell RNA sequencing (scRNA-seq) have provided the opportunity to identify disease-related cell subsets and state in tissue and blood samples, which is different from traditional RNA-seq. Through scRNA-seq, the gene expression of single cell can be measured, and the specific cellular subsets and cell-type-specific pathways involved in the pathogenesis of disease could be detected.

In recent years, scRNA-seq has been used to analyze some autoimmune diseases, including rheumatoid arthritis (RA) and systemic lupus erythematosus (SLE) (14, 15). However, scRNA-seq was rarely applied in pSS. In this study, we collected PBMCs from five patients with pSS and five healthy controls to perform scRNA-seq. We sought to map the cellular landscapes and match the circulating blood cells of patients with pSS to help dissect disease heterogeneity among patients and to identify the underlying cellular and molecular events related to disease outcomes and responses to treatment.

## Material and Methods

### Acquisition of the Study Sample

This study was approved by the Ethics Committee of the Shenzhen People’s Hospital, China (LL-KY 2019514), and all donors signed a written informed consent. Five patients with pSS and five healthy controls were recruited in this study; all patients with pSS were from the Department of Rheumatology and Immunology, Shenzhen People’s Hospital, China and diagnosed with pSS according to the 2016 American College of Rheumatology(ACR)/European League Against Rheumatism (Eular) classification criteria for pSS (16) **(** **Supplementary Table 1**). 8 ml of peripheral blood was collected from each sample, and PBMCs were isolated using density gradient centrifugation with Ficoll-Hypaque. We washed them with chilled PBS, counted the PBMCs, and stored them on ice for subsequent experiments.

### Single-Cell Capture, Library Construction, and Sequencing

The brief introduction of each step are as follows: (1) Based on the latest chromium™ Single Cell 3′ Solution system of 10× Genomics, Gel bead with barcode and primer and a single cell were wrapped in oil drops to form GEM (Gel bead in emulsion). GEM refers to the mixture of gel beads containing barcode, cells, and reagents wrapped in oil droplets. The GEM was recovered and purified for subsequent experiments. (2) cDNA formation and amplification: the gel beads in the GEM were dissolved, and the cells were lysed to release the mRNA, and the barcoded cDNA used for sequencing was generated by reverse transcription; after the liquid oil layer was destroyed, the cDNA amplification reaction was performed, and the library was constructed after the quality inspection was qualified. (3) Library construction: firstly, the cDNA was digested into fragments of about 200–300 bp, and then the library construction process of traditional second-generation sequencing such as sequencing adapter P5 and sequencing primer R1, and finally the DNA library was obtained by PCR amplification. (4) Sequencing: High-throughput sequencing of the library was performed using the paired-end sequencing model of Illumina sequencing platform.

### Clustering Cells

Using the 10× Genomics official analysis software Cell Ranger (https://support.10xgenomics.com/single-cell-geneexpression/software/overview/welcome), the original data was filtered, compared, quantified, identified, and recovered cells, and finally, the gene expression matrix of each cell was obtained. The process of clustering cells was as follows: Seurat is a popular R package, which was developed as a clustering tool for scRNA-seq data, and it can perform quality control, analysis, and exploration of scRNA-seq data. After removing low-quality cells, we first normalize the expression of the data, a global-scaling normalization method “LogNormalize” of Seurat software was performed. Then the PCA (principal component analysis) analysis was performed using the normalized expression value. Using Jackstraw substitution test algorithm, we select the most significant (*P* < 1e-5) principal component (PC) from the PCA analysis results for subsequent clustering and cluster analysis. Seurat implements a graph-based clustering method. This method has been used in recent manuscripts, such as graph-based clustering approaches to scRNA-seq data—SNN-Cliq (17) and CyTOF data—PhenoGraph (18). In order to cluster the cells, the modularity optimization techniques —SLM was applied (19). Seurat continues to use t-SNE (t-distributed Stochastic Neighbor Embedding) (20) as a powerful tool to visualize and explore these datasets.

### Antibodies and Flow Cytometric Analysis

**Figure 2:**
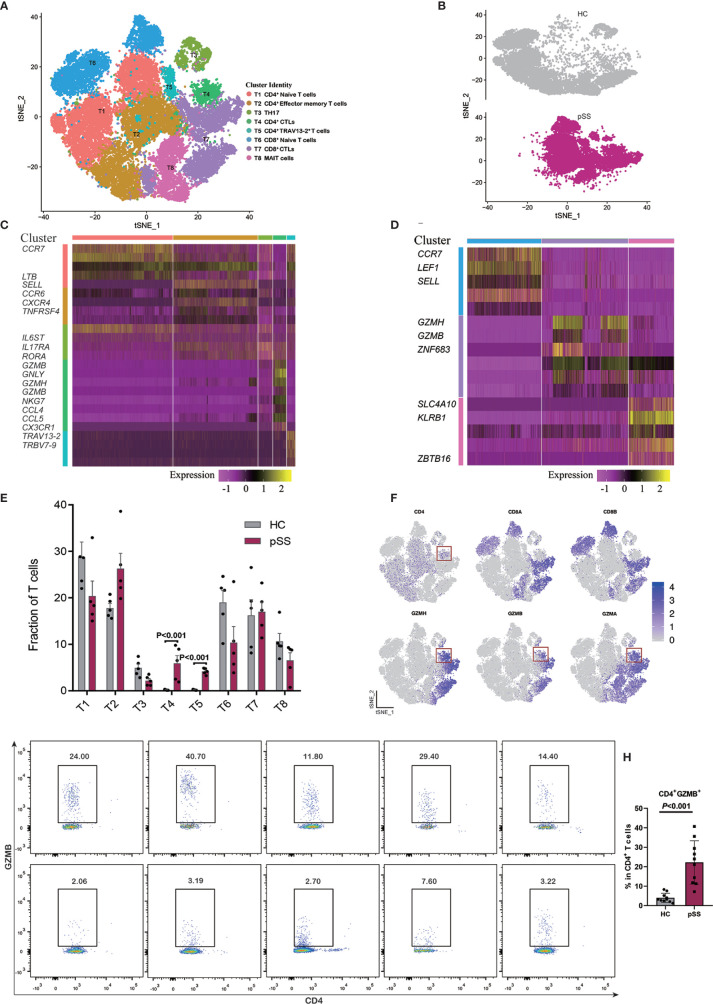
Identifying T cell subpopulations. **(A)** t-SNE visualization of 33,081 T cells from healthy controls (HCs) (n = 5) and patients with pSS (n = 5), including five CD4^+^ T cell clusters, three CD8^+^ T cell clusters. **(B)** Annotating condition of HCs (n = 5) and patients with pSS (n = 5). **(C)** Heat map of the five CD4^+^ T cell clusters (T1–T5). **(D)** Heat map of the three CD8 T cell clusters (T6–T8), rows represent selected differentially expressed signature genes in each cluster, and different clusters are exhibited in the rows. **(E)** Fractions of T cell subpopulations in HCs (n = 5) and patients with pSS (n = 5), the results calculated by multiple t tests, the differences were considered significant if the p value was less than 0.05. **(F)** Expression of selective marker genes for CD4^+^ CTLs (T4), and the cell positions are in the t-SNE plot of panels **(A, G)** The profiles of patients with pSS (pSS1-5) and healthy controls (HC1-5). Cells gated on CD3^+^ were profiled using CD4 (x axis) and GZMB (y axis), CD4^+^ CTLs are on top right corners. **(H)** Percentages of CD4^+^ GZMB^+^ T cells among the CD4^+^ T cells of the 10 patients with pSS and 10 healthy controls in **(G)**; the results were calculated by t tests, and the differences were considered significant if the p value was less than 0.05.

10 patients with pSS and 10 healthy controls were recruited (**Supplementary Table 2**), and the whole blood were incubated with antibody and then treated with Red blood cell lysis buffer. Monoclonal antibodies specific for human CD3 (UCHT1), CD4 (RPA-T4), and GZMB (GB11) were purchased from BD Pharmingen. For intracellular staining, cells were fixed and permeabilized with IntraPrep Permeabilization Reagent (Beckman Coulter) according to the manufacturer’s protocols. Cells were analyzed using FACS Cano II. The percentage of CD4^+^ GZMB^+^ T cells was calculated by t tests, and the differences were considered significant if the *P* value was less than 0.05 (**Figure 2H**).

### RNA Extraction and RT-qPCR

**Figure 3:**
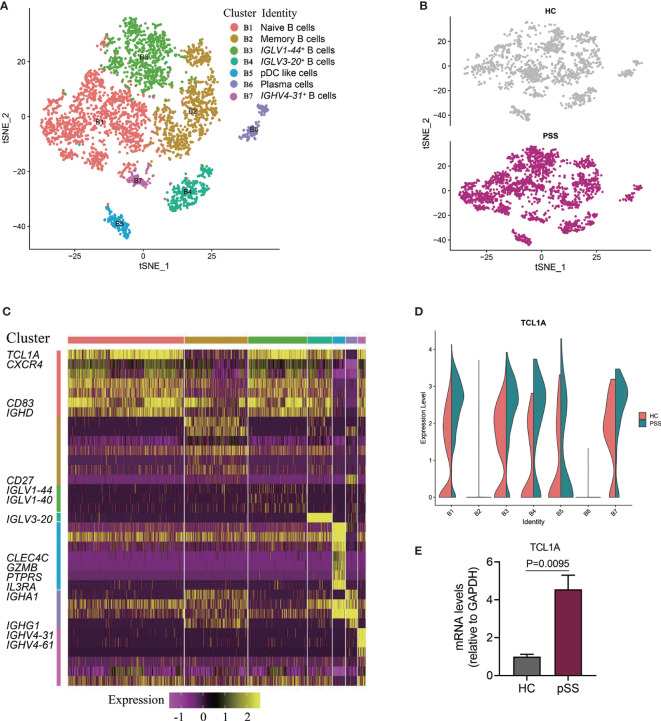
Identifying B cell subpopulations. **(A)** Two-dimensional t-SNE visualization of 3,912 B cells from HCs (n = 5) and patients with pSS (n = 5). **(B)** Annotating condition of HCs (n = 5) and patients with pSS (n = 5). **(C)** Heat map of seven B cell clusters. Columns represent selected differentially expressed signature genes in each cluster, and different clusters are exhibited in the rows. **(D)** The results were calculated by the likelihood-ratio test, and the differences were considered significant if the p value was less than 0.05; violin plots showing the differential expression of TCL1A in each cluster of B cells for the HCs (n = 5) and patients with pSS (n = 5); the *P* value < 0.01 in cluster B1, B2, B3, and B4. **(E)** The expression of TCL1A in B cells of the six patients with pSS and four healthy controls were validated by RT-qPCR.

Six patients with pSS and four healthy controls were recruited (**Supplementary Table 3**); 8 ml of peripheral blood was collected from each sample, and PBMCs were isolated using density gradient centrifugation with Ficoll-Hypaque. Then B cells were isolated from PBMCs by CD19 positive selection using MACS magnetic beads (Miltenyi). The RNA was extracted from B cells using RNA extraction kit (RNeasy Micro Kit), and total RNA was reversed transcribed into cDNA. The cDNA then was used for Quantitative real-time PCR (RT-qPCR) analysis in a StepOne plus machine (Life Technology). The expression of TCL1A in B cells was calculated by t tests, and the differences were considered significant if the P value was less than 0.05 (**Figure 3E**).

The primer sequences for TCL1A were as follows: forward, AGTTACGGGTGCTCTTGC; Reverse, TCGGTATCGTCCATCAGG.

### Differentially Expressed Gene Analysis

Differentially expressed gene analysis for each cluster: we used the likelihood-ratio test (21) to find the differential expression for a single cluster, compared to all other cells. Differentially expressed genes are as the following criteria: (1) *P*-value ≤ 0.01. (2) log2 FC ≥ 0.36. log2 FC means log fold-change of the average expression between the two groups. (3) The percentage of cells where the gene is detected in specific cluster >25%. Using this method, we analyzed differentially expressed genes for 19 clusters of PBMCs between HCs and pSS.

### Gene Ontololgy and Kyoto Encyclopedia of Genes and Genomes Pathway Enrichment Analysis for Different Cell Types

We analyzed differentially expressed genes of multiple cell types including T cells, NKs, B cells, CD14^+^ monocytes, macrophages, and DCs between patients with pSS and healthy controls by likelihood-ratio test, then the selected upregulated expressed genes were mapped to each term in the GO database (http://www.geneontology.org/); differently enriched GO term was calculated by hypergeometric test. The calculated *P*-value was corrected by FDR, and *P*-value ≤0.05 was considered to be statistically significant. KEGG pathway was calculated in the same way as GO.

## Results

### Generation of Transcriptomic Data From Peripheral Blood Mononuclear Cells

**Figure 1:**
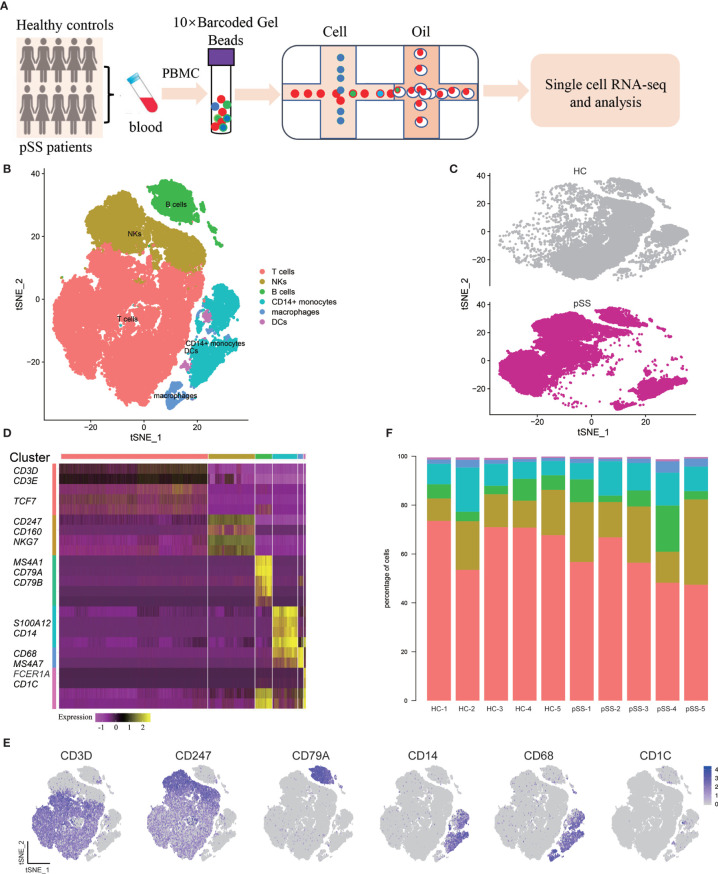
Single-cell RNA-seq design and initial analysis. **(A)** Schematic representation of experimental strategy. **(B)** Two-dimensional t-SNE visualization of 57,288 cells from healthy controls (HCs) (n = 5) and patients with pSS (n = 5). **(C)** Annotating condition of HCs (n = 5) and patients with pSS (n = 5). **(D)** Heat map of major cell types. Columns represent selected differentially expressed signature genes in each cluster, and different clusters are exhibited in the rows. **(E)** Expression of selective marker genes for six major cell types, and the cell positions are in the tSNE plot of Figure B. **(F)** cellular composition of each sample, the colors represent different cell types.

Using the 10× Genomics platform, we performed scRNA-seq on PBMCs from five patients with pSS and five healthy controls (**Figure 1A**). After quality control, poor-quality cells were filtered out, and a whole-transcriptome database of 57,288 cells from five healthy controls and five patients with pSS were analyzed. Major immune cell types, including T cells, natural killer (NK) cells, B cells, and monocytes were classified and identified using PhenoGraph clustering (18) (**Figures 1B, C**, **Supplementary Figure 1**). Differentially expressed genes between cell types, which were identified based on mean expression and covariance patterns, were analyzed, and the selected top genes that were unique to each cluster based on the average log fold-change showed a high degree of heterogeneity among the clusters. T cells showed specific expression of *CD3D* and *CD3E* that distinguished them from other clusters (22). NK cells were identified by the lack of *CD3D* and *CD3E* and high expression of *NKG7* and *CD247* (23), and B cells were characterized by high expression of *MS4A1* (*CD20*), *CD79A*, and *CD79B* (22). We also identified three major subsets of monocytes, *CD14* ^+^ monocytes, macrophages, and DCs. The enrichment of *CD14*, *SL00A8*, and *SL00A9* is known in *CD14* ^+^ monocytes (24); in addition, macrophages expressed high level of *CD68* and *MS4A7* (25), and DCs are defined by *CD1C* (**Figures 1D, E** **)** (26). We found some degree of difference in the immune composition of each individual sample; for example, the DC fractions constituted less than 1%, and T cells constituted 47–73% (**Figure 1F**).

### Analysis of CD4+ and CD8+ T Cells

T cells can coordinate adaptive immunity by producing cytokines and effector molecules. To reveal the internal structure and functional subtypes in all T cell populations, unsupervised clustering of all T cells was performed (27). We merged the transcriptomic data of 10 donors (five healthy controls and five patients with pSS), and eight different clusters emerged, including five CD4^+^ T cell (T1–T5) and three CD8^+^ T cell (T6–T8) clusters based on distinct signature genes (**Figures 2A, B**, **Supplementary Figure 2**). The markers associated with the naive T cell, such as *CCR7*, *LTB*, and *SELL* (28), were highly expressed in the T1 cluster. Cluster T2 was defined as effector memory CD4^+^ T cells (TEM) with the high expression of *CXCR4*, *TNFRSF4*, and *CCR6*, and this cluster was associated with the effector functions of T cells, while cluster T3 expressed *RORA*, *IL6ST*, and *IL17RA*, suggesting a T_H_17 cell identity (29). Clusters T4 and T5 were specifically expanded in the CD4^+^ T cell composition of each pSS patient (**Figure 2E**); cytotoxicity associated genes (*GZMH*, *GZMA*, and *GZMB*) were expressed in cluster T4 (**Figure 2F**), which we defined as CD4^+^ CTLs. To validate this finding, we then choose one of these cytotoxic genes to perform the experiment by using flow cytometry. Consistently, a marked expansion of CD4^+^ GZMB^+^ T cells were confirmed in patients with pSS (**Figure 2G**), and the percentages of CD4^+^ GZMB^+^ T cells in the CD4^+^ T cell populations were significantly higher in the pSS than in the healthy controls (*P<*0.001) (**Figure 2H**). T cell receptor alpha and beta chain variable genes with *TRAV13-2* and *TRBV7-9* were enriched in cluster T5 (**Figure 2C**), and this polymorphism in T cell receptor (TCR) genes could determine the occurrence of a pathogenic response. Cluster T6 was characterized as naive CD8^+^ T cells with the high expression of *CCR7*, *LEF1*, and *CD27*. Cluster T7 exhibited high levels of cytotoxic genes, including *GZMH*, *GZMB*, and *ZNF683*, suggesting the identity of activated CD8^+^ CTLs. The third cluster of CD8^+^ T cells was marked by differentially expressed genes, including *SLC4A10*, *KLRB1*, and *ZBTB16*, a mucosal-associated invariant T cells (MAIT)-like identity (**Figure 2D**) (30).

### Analysis of B Cell Subsets

Previous studies have assessed B cell subpopulations in the salivary glands and peripheral blood of patients with pSS, and one study found increased number of plasmablasts and memory B cells in patients with SS who had lymphoma (31). In our study, we analyzed a subpopulation of B cells and differentially expressed genes by scRNA-seq; B cells were defined (**Figure 1B**), and then we further analyzed 3,912 cells located in B cells from five patients with pSS and five healthy controls. Seven different B cell clusters were found in the PBMC samples, and four B cell clusters including naive B cells (B1), memory B cells (B2), plasmacytoid DCs (B5) and plasma cells (B6) were identified (**Figures 3A, B**, **Supplementary Figure 3**). Cluster B1 expressed naive B cells related genes (*CXCR4*, *CD83*, and *IGHD*) (15), and *CD27* was expressed in clusters B2 and B6. Cluster B2 was similar to memory B cell (32), and cluster B6 expressed high levels of immunoglobulin genes (*IGHA1*, *IGHG1*, and *IGLC2*), which defined them as plasma cells. Cluster B5 exhibited the expression of *CLEC4C*, *GZMB*, *PTPRS*, and *IL3RA*, which is similar to the phenotype of pDC-like cells (14). Clusters B3, B4, and B7 mainly expressed immunoglobulin (Ig) encoded by the Ig light/heavy-chain-variable-region genes (IGLV/IGHV) (**Figure 3C**), and these immunoglobulin genes serve as B cell receptors (BCRs) that bind to specific antigens, contributing to the clonal expansion and differentiation of B lymphocytes, which is associated with disease activity and autoantibodies production.

T-Cell Lymphoma1A (TCL1A) is an oncogene that has an important role in lymphomagenesis and acts as a coactivator of AKT kinases. The expression of TCL1A is deregulated in lymphocytic leukemia (B-CLL) and most lymphomas, which involve several signaling pathways, such as the phosphatidylinositol 3 kinase (PI3K) and nuclear factor-kB (NF-kB) pathways (33). In our study, *TCLIA* was broadly expressed in most B cells (**Figure 3B**); compared with HCs, we found that *TCLIA* expression was significantly upregulated in multiple B cell subpopulations from the patients with pSS (**Figure 3D**).To ascertain the expression of TCL1A in B cells, we performed RT-qPCR, and the result indicated that the expression level of TCL1A is higher in patients with pSS than that in healthy controls (***P* = 0.0095,** **Figure 3E**).

### Differentially Expressed Gene (Upregulation) Analysis in Each Cell Subtype From the Patients With Primary Sjögren’s Syndrome

**Figure 4:**
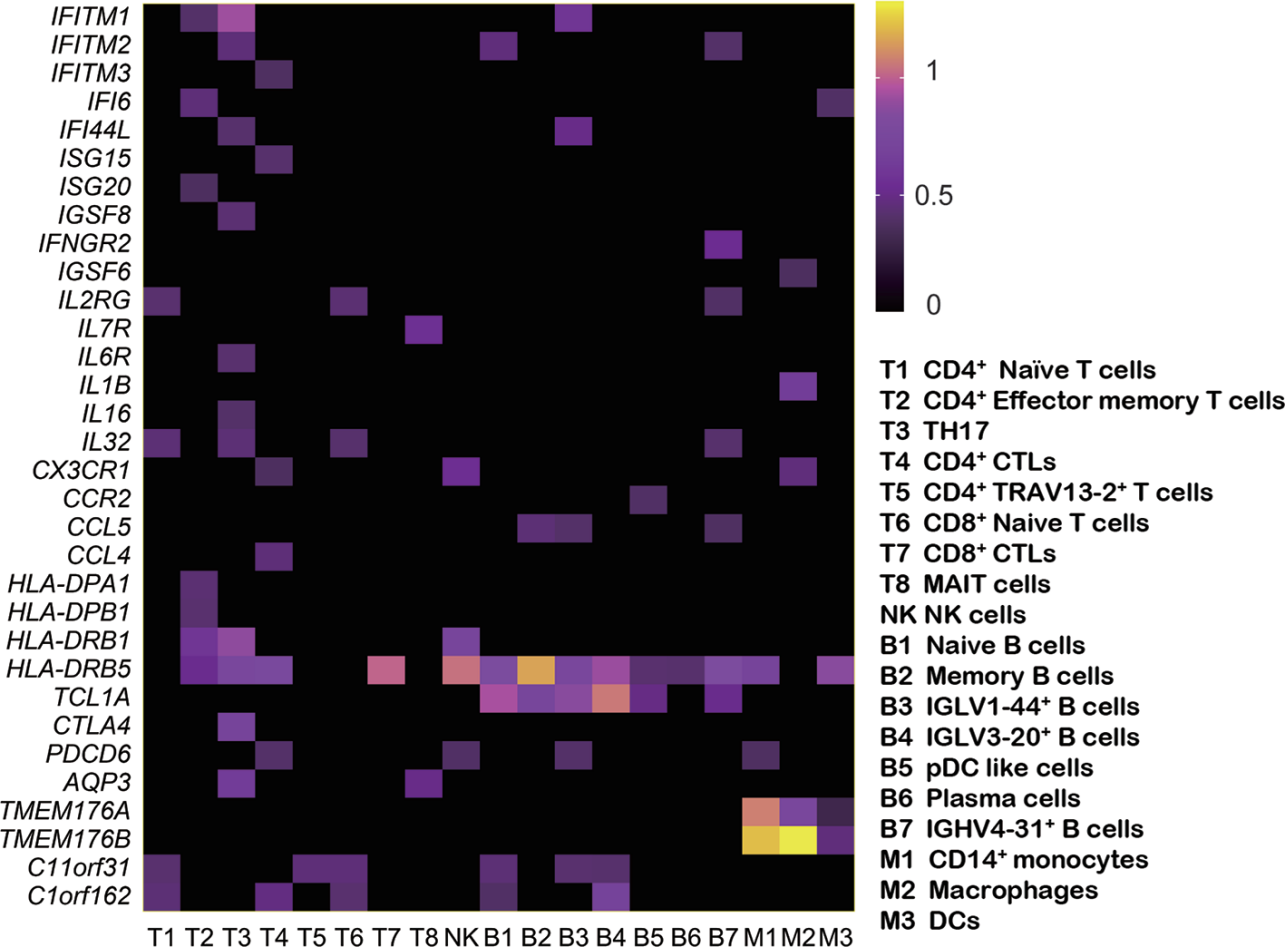
Expression of upregulated genes in patients with pSS. Based on comparison with the HC samples, the heat map shows the upregulated genes (log2 FC ≥ 0.36 and *P*-value ≤ 0.01) in each pSS cluster. Rows represent each cluster over all cells, and columns represent each gene in each cluster. The rows and columns are clustered based on Euclidean distance.

Using the likelihood-ratio test (21), we analyzed differentially expressed genes for each cell cluster and chose upregulated genes (log2 FC ≥ 0.36 and *P*-value ≤ 0.01) in pSS (**Figure 4**). Several foregone and expected genes were found. First, a series of interferon response genes were upregulated in most cell subsets, including interferon inducible protein (IFI) *IFI6*, *IFI16*, and *IFI44L*; the interferon-induced transmembrane proteins (IFITMs) *IFITM1*, *IFITM2*, and *IFITM3*; and the IFN-stimulated genes (ISGs) *ISG15*, and *ISG20*. The levels of cytokines (*IL-32* and *IL-16*) and chemokines (*CCL4*, *CCL5*, and *CX3CR1*) also increased. Chromosome X Open Reading Frame 21 (CXorf21) has been observed to be a susceptibility gene in SLE (34), and here, Chromosome 11 Open Reading Frame 31 (*C11orf31*) and Chromosome 1 Open Reading Frame 162 (*C1orf162*) were broadly expressed in subtypes of T and B cells and monocytes. *TMEM176A* and *TMEM176B*, which were suggested to inhibit DC maturation in chronic spinal cord injury (35), were expressed in three subtypes of monocytes. Genome-wide association studies (GWASs) reported that the genetic locus of most MHC regions, such as *HLA-DQB1*, *HLA-DRA1*, *HLA-DQA1*, were associated with pSS (36). Our study also identified *HLA-DQB1* (T cells and monocytes). In addition, *HLA-DPA1*, *HLA-DPB1* (T cells), and *HLA-DRB1* (T cells and NK cells) were expressed in T cells, and *HLA-DRB5* was the most significantly expressed gene in each cell type of pSS. Cytotoxic T-lymphocyte-associated protein 4 (*CTLA4)* (T_H_17), *PDCD6*, and *AQP3* were also identified. In summary, our study not only confirmed genes previously reported to be associated with pSS, but also found additional signature markers for different cell types.

### Disease-Associated Pathways Revealed in Patients With Primary Sjögren’s Syndrome

**Figure 5:**
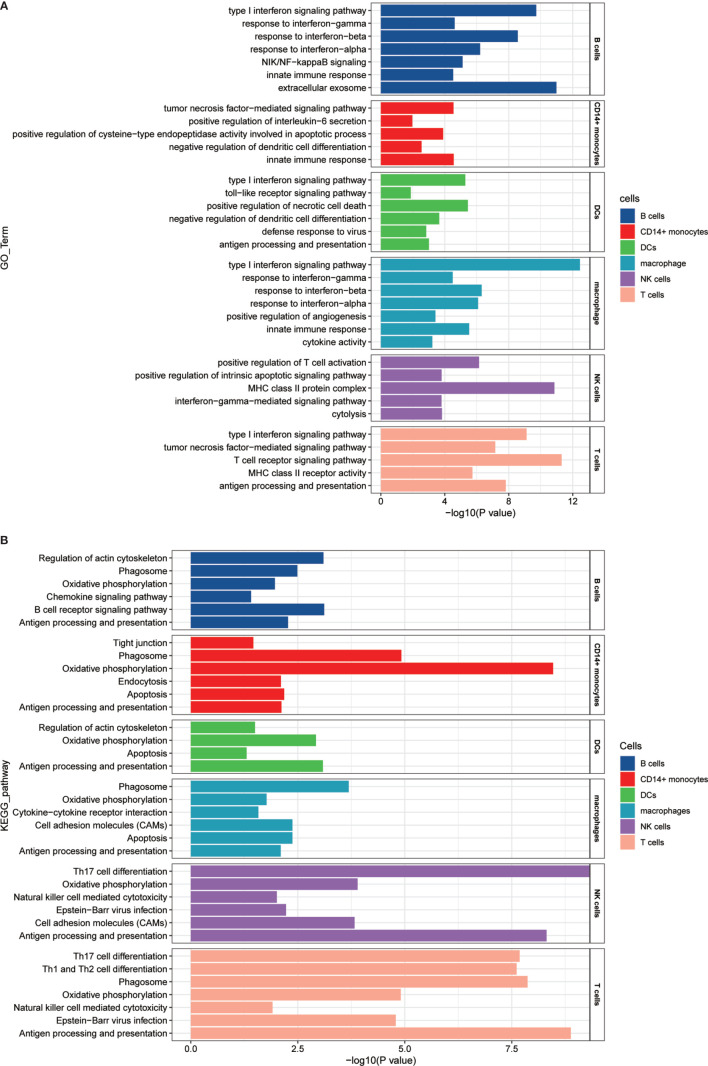
GO and KEGG pathway enrichment analysis of the upregulated genes in patients with pSS. **(A)** GO pathway enrichment analysis of upregulated genes was performed for each cell type in the pSS samples, and selected pSS-associated pathways in each cell type are shown. **(B)** KEGG pathway enrichment analysis of upregulated genes was performed for each cell type in the pSS samples, and selected pSS-associated pathways in each cell type are shown. The calculated p-value was determined through FDR correction, and we used FDR ≤0.05 as the threshold. The length of −log10 (*P*-value) determined the degree of enrichment from the least significant to the most significant.

To analyze whether there are cell subset-specific pathways in patients, we performed Gene Ontology (GO) pathway analysis for upregulated genes in the patients. The IFN signature is the typical characteristic of several autoimmune diseases, such as SLE, pSS, and RA, which positively participates in inflammatory reaction. In this study, we also confirmed this association (37). Increased type I IFN (mainly IFNα and IFN*β*) and type II IFN (IFN*γ*) signaling was activated in most cells, and IFN-associated genes, including IFITM3, IFITM2, IFITM1, and XAF1, had a significant overlap between types I and II. Tumor necrosis factor (TNF) family signaling and antigen processing and presentation pathways were upregulated. Cell subset-specific pathways, such as negative regulation of DC differentiation by TMEM176A and TMEM176B, were observed in monocytes (**Figure 5A**). We also performed Kyoto Encyclopedia of Genes and Genomes (KEGG) pathway analysis, and several immune-associated pathways, such as apoptosis, phagocytosis and oxidative phosphorylation, were activated. T_H_1, T_H_2, and T_H_17 cell differentiation signaling pathways were enriched in T cells **(** **Figure 5B** **)** and regulated by IL2RG, IL4R, and HLA-DRB5, indicating the involvement of T_H_1, T_H_2, and T_H_17 cells in pSS. This analysis identified several upregulated immune-related pathways, contributing to the understanding of disease pathogenesis. Therefore, targeting pathways may be an efficient therapeutic strategy for treating pSS.

## Discussion

Using scRNA-Seq to study PBMC samples from patients with pSS and healthy controls, we revealed the complexity of immune cell populations and recognized the considerable variation in patients with pSS. Our analysis focused on T cells and B cells. Here, we found major cell groups and disease-specific subsets and then identified several signatures and essential inflammatory pathways associated with the disease.

Our transcriptomic analysis created a detailed overview of the T cell populations, and five CD4^+^ and three CD8^+^ T cell clusters were identified. Two CD4^+^ T cell subpopulations, CD4^+^ CTLs and TCR variable genes CD4^+^ T cells, were found to specifically expand in the patients with pSS. For the CD4^+^ CTL subpopulation, there was specific amplification of several transcripts linked to the cytotoxic function of CD8^+^ T lymphocytes such as GNLY, GZMB, and NKG7. Traditionally, the effect of CD8 CTLs is to eliminate target cells using cytotoxic molecules, and the function of CD4 T cells, generally called helper T cell, is to regulate the immune responses *via* various cytokines. However, in recent years, the presence of CD4^+^ CTLs has been reported in various diseases. CD4^+^ CTLs were not only observed when infected with viruses, including human immunodeficiency virus (HIV) and cytomegalovirus (CMV) (38), but also were found at the site of inflammation of several autoimmune diseases, such as RA (39), IgG4-related disease (40), and systemic sclerosis (41). Moreover, in some autoimmune diseases, such as RA (42), and Granulomatosis with Polyangiitis (43), research revealed the correlation of CD4^+^ CTLs expansion and CMV infection, indicating that CMV infection may play a role in CD4^+^ CTL-mediated damage. Besides, we also need take into consideration that patients with autoimmune diseases have increased susceptibility to infection.

In our study, the occupancy of CD4^+^ CTLs in pSS measured by scRNA-Seq was significantly higher than that in healthy controls. However, patients in the pSS group presented herein were older than healthy controls. To address this problem, we performed validation experiment with an age-matched control group, and the FACS results are consistent with the scRNA-seq results (**Figure 2H**, **Supplementary Table 2**). In healthy controls, the fractions of CD4^+^ CTLs are rare, consistent with previous studies (44, 45).We also studied the correlation between the percentage of CD4^+^ CTLs and clinical characteristics such as ESR (erythrocyte sedimentation rate), anti-SSA positive, and ESSDAI (the European League Against Rheumatism Sjögren’s syndrome disease activity index), but no significant correlation was found.

Further analyses of scRNA-Seq indicated that the expression of chemokines, such as CCL5, CCL4, and CX3CR1, is upregulated in CD4^+^ CTLs **(** **Figure 2C** **)**. A previous study in RA showed that the CD4^+^ CTL existed in peripheral blood and synovium; upregulated chemokine receptor CX3CR1 could drive CD4^+^ CTLs to synovium due to the expression of CX3CR1 ligand, fractalkine (CX3CL1) in synoviocytes (46). In pSS, two studies also revealed that the CX3CR1 ligand, fractalkine (CX3CL1), was upregulated in the salivary glands (47, 48). Therefore, we speculated that CD4^+^ CTLs may migrate to the salivary glands driven by chemokines. Collectively, all the information suggests that the subpopulation of CD4^+^ CTLs may play an important role in inflammation of autoimmune disease.

B cells play a central role in the pathogenesis of pSS through B cells’ overactivation, and therapeutic strategies, by deleting B cells or inhibiting B cells’ signal to impair B cell activation and differentiation, have achieved certain progress (49). A 2016 study performing immunophenotyping by time-of-flight (CyTOF) provided new insights into the involved B cell subpopulations, such as plasmablasts and CD27^+^ memory B cells, in patients with pSS (50). Another study indicated the importance of plasma cell infiltration in patients with pSS with interstitial nephritis (51). Our findings offered a detailed map of B cells created by single-cell transcriptomic analysis, revealing different subpopulations of B cells defined by uniquely expressed markers, including naive B cells, memory B cells, and plasma cells, which may be helpful for targeted therapy of B cell subsets. IGLV/IGHV genes associated with B cell subsets were also found, and these immunoglobulins participate in the identification of antigens and commonly determine the antigen specificity of BCR, which requires additional BCR repertoire analysis. In patients with pSS, B-cell hyperactivity is also associated with an increased risk of developing B-cell lymphoma, which is 15 to 20 times higher than that of healthy people, and pSS-associated lymphomas are mostly low-grade B-cell non-Hodgkin’s lymphoma (NHL) (52, 53). In China, the risk of NHL in patients with pSS is 48.1 times higher than that in healthy people (54). A study has confirmed increased expression of TCL1A in NHL (55). Our scRNA-seq and RT-qPCR results also showed significant upregulation of TCL1A in B cells, implying a potential risk of NHL in patients with pSS studied herein. However, studies on TCL1A in pSS are rare, requiring further studies to explore the mechanism of TCL1A in the disease.

Several genome-wide association studies (GWASs) have established associations between pSS and susceptibility genes. Our study observed upregulation of IFN, cytokine, and chemokine expression. We also identified several potential signatures, although with poor annotation for pSS, such as CTLA4, which exerts an inhibitory effect on T cells by competitively inhibiting binding of the common ligand B7 (CD80/CD86) to CD28. The therapeutic approach of using antibodies to block CTLA4 has been used in many cancer types and demonstrated unprecedented efficacy (56). Abatacept (a CTLA4 agonist) has received approval for the treatment of RA, and it is proved to be effective (57). A previous study demonstrated a substantial increase in the expression of the negative regulatory molecules PD-1 and CTLA-4 in Pss (58). In our study, CTLA4 was uniquely expressed in the T_H_17 cells of patients with pSS, and its potential mechanism deserves further exploration. Water movement was previously reported to be involved in exocrine secretion, and aquaporins (AQPs) may lead to fluid secretion in the exocrine glands (59). AQP5 has been shown to play an important role in the salivary secretion process (60). In our study, the upregulation of AQP3 is identified and may play a potential role in pSS. The exploration of susceptibility genes is urgently needed to develop novel therapeutic targets for disease state-specific treatment.

It has been reported that innate immune cells producing type I IFNs play a vital role in systemic autoimmunity, and type I IFNs are produced primarily by pDCs. Activated pDCs have been detected in minor salivary gland biopsies of patients with pSS (61, 62). Consistent with previous reports, we also identified activated type I IFN signaling pathways in multiple types of PBMCs. In addition, the type II IFN signaling pathway was also activated, and our study can offer a reference for targeted therapy focused on IFN. We also detected T_H_1, T_H_2, and T_H_17 cell differentiation signaling in pSS, which can lead to the activation of adaptive immunity. A previous study showed that T_H_1 and T_H_17 cells play a role in the initial stage of SS, but T_H_2 and T_FH_ cells take a dominant role in disease progression (63), suggesting T_H_ cells affect the pathogenesis of pSS. Our data provide an opportunity to target these immune-associated signaling pathways.

Although limited sample size and lack of diversity in disease presentation impede subsetting of patients in our research, our data confirms that scRNA-seq is feasible and provides a large amount of data, which exhibited marked complexity. The application of single-cell sequencing technologies helps us not only define major cell populations and novel cell subpopulations, but also reveal molecular signatures relevant to disease, thereby offering new insights into therapeutic strategies.

## Data Availability Statement

The raw and processed data from single-cell sequencing in this study have been deposited with the Gene Expression Omnibus under accession number GSE157278.

## Ethics Statement

The studies involving human participants were reviewed and approved by the Ethics Committee of the Shenzhen People’s Hospital, China (LL-KY 2019514). The patients/participants provided their written informed consent to participate in this study. Written informed consent was obtained from the individual(s) for the publication of any potentially identifiable images or data included in this article.

## Author Contributions

MY and YD designed and supervised the study. XH and SM collected the clinical samples, performed the sequencing experiments, wrote the original manuscript, and analyzed the data. DT and TW performed the sequencing experiments and revised the manuscript. LD, HY, HL, and DL helped analyzed data. All authors contributed to the article and approved the submitted version.

## Funding

This project was supported by the National Natural Science Foundation of China (Grant No. 81671596 and 81771747) and the Natural Science Foundation of Guangdong Province (Grant No. 2017A030313475).

## Conflict of Interest

The authors declare that the research was conducted in the absence of any commercial or financial relationships that could be construed as a potential conflict of interest.

## Supplementary Material

Supplementary Figure 1 t-SNE visualization for each sample.

Supplementary Figure 2 t-SNE visualization of T cells for each sample.

Supplementary Figure 3 t-SNE visualization of B cells for each sample.
