# Supplementary figures and images for: Single-Cell RNA Sequencing Reveals the Expansion of Cytotoxic CD4+ T Lymphocytes and a Landscape of Immune Cells in Primary Sjögren’s Syndrome
Source: Front Immunol. 2021 Feb 2;11:594658. doi: 10.3389/fimmu.2020.594658 (PMC7884617; doi:10.3389/fimmu.2020.594658)

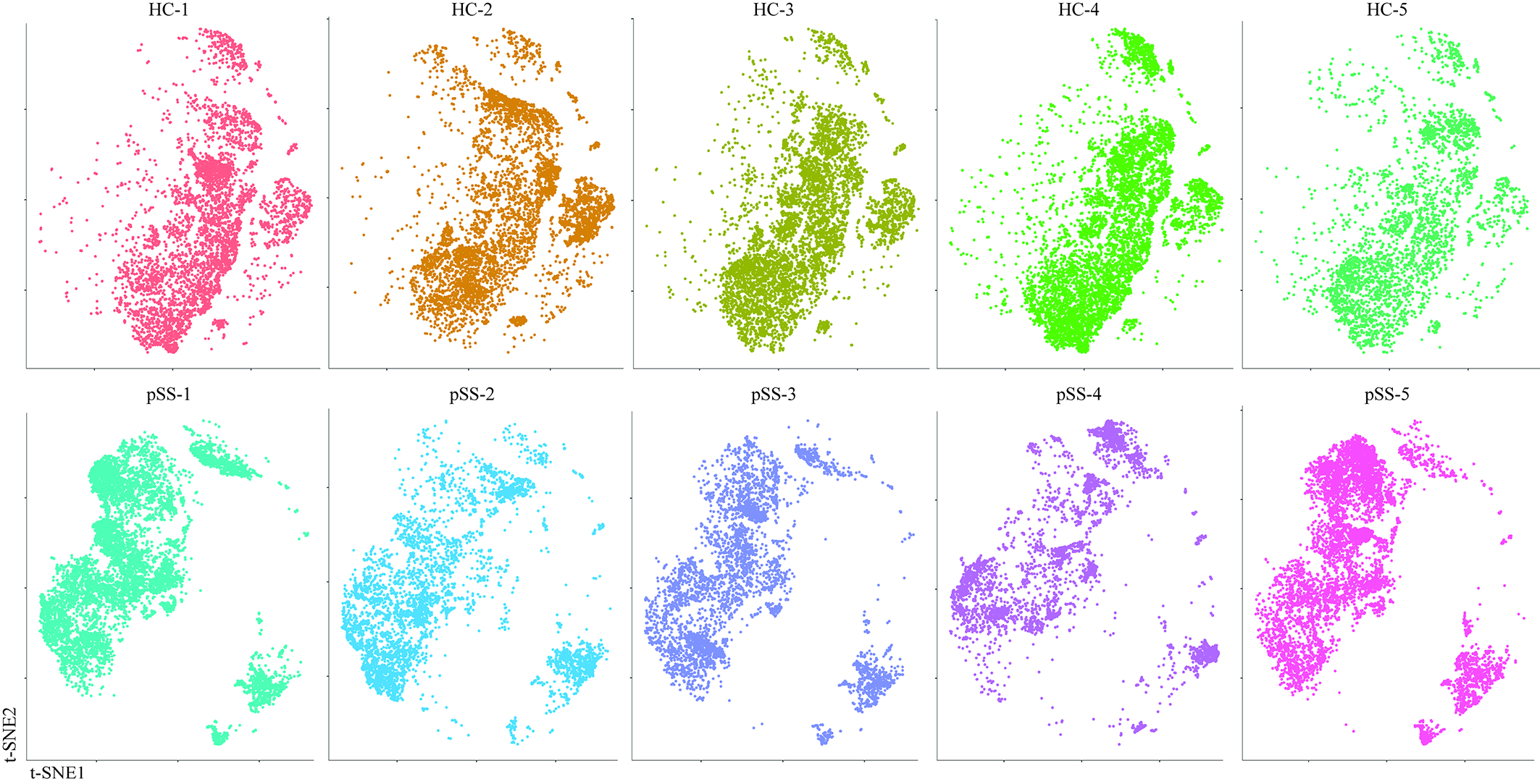

Supplement: Supplementary Figure 1 — t-SNE visualization for each sample. [file Image_1.tif]

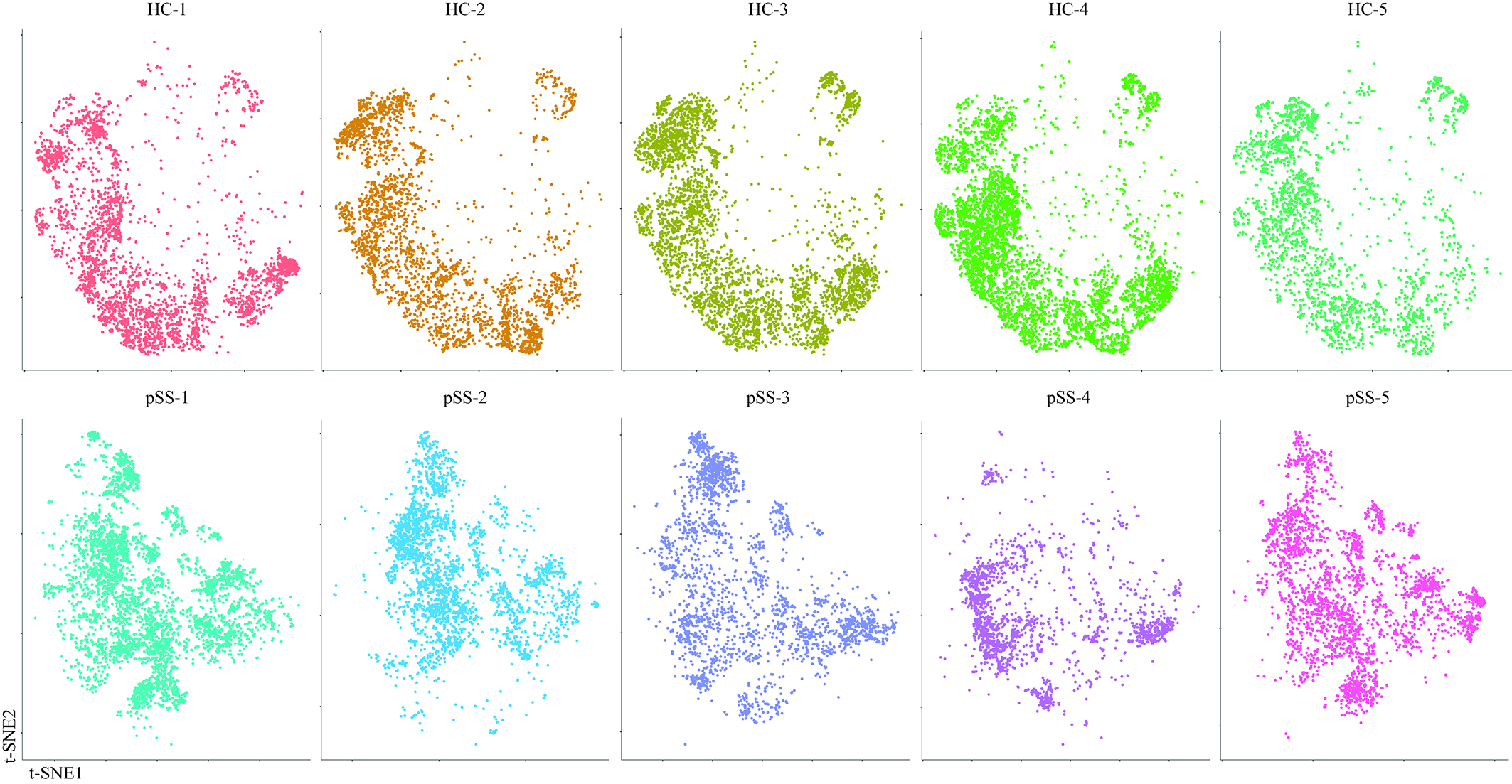

Supplement: Supplementary Figure 2 — t-SNE visualization of T cells for each sample. [file Image_2.tif]

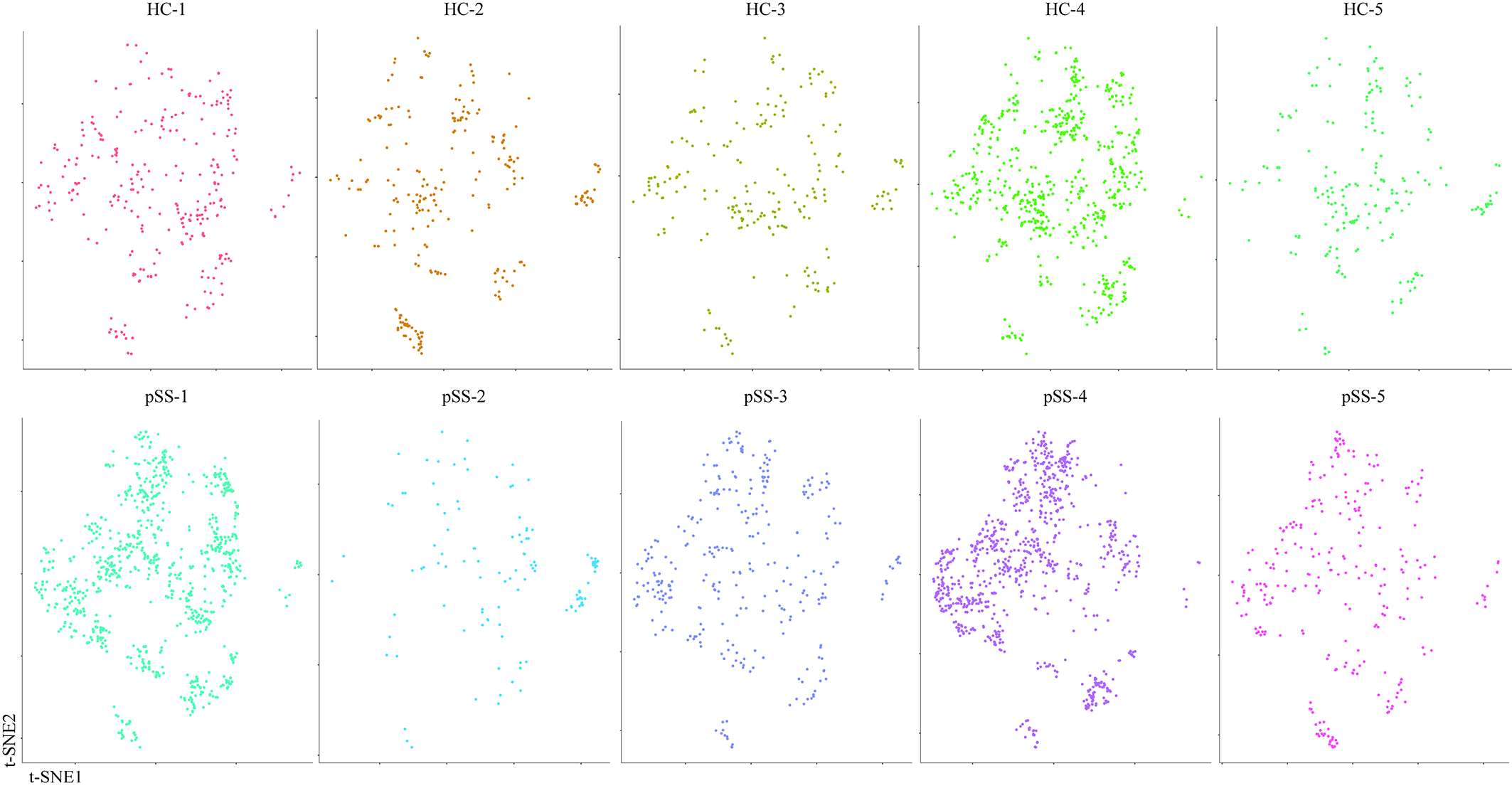

Supplement: Supplementary Figure 3 — t-SNE visualization of B cells for each sample. [file Image_3.tif]
