# Supplementary material for: Single-Cell RNA Sequencing Reveals the Expansion of Cytotoxic CD4+ T Lymphocytes and a Landscape of Immune Cells in Primary Sjögren’s Syndrome
Source: Front Immunol. 2021 Feb 2;11:594658. doi: 10.3389/fimmu.2020.594658 (PMC7884617; doi:10.3389/fimmu.2020.594658)
Supplement: Supplementary file 4 [file Table_1.docx]

**SUPPLEMENTARY MATERIALS**

Manuscript: **Single-cell RNA Sequencing reveals the expansion of Cytotoxic CD4^+^ T lymphocytes and a landscape of immune cells in primary Sjögren’s syndrome**

Authors: Xiaoping Hong^1,2,^*, Shuhui Meng^2,^*, Donge Tang^2^, Tingting Wang^2^, Liping Ding^2^, Haiyan Yu^2^, Heng Li^2^, Dongzhou Liu^2^, Yong Dai^2#^, Min Yang^1#^

LIST OF SUPPLEMENTARY MATERIALS

1. Supplementary Table1-3

**Clinical and laboratory features of study participants**

Patient’s clinical and laboratory features were analyzed. All patients fulfilled 2016 ACR/Eular classification criteria for pSS. The EULAR SS disease activity index (ESSDAI) score of pSS patients were calculated.

**Supplementary Table-1. Clinical and laboratory features of patients with pSS and Healthy controls (HC) studied for scRNA-seq**

| Clinical characteristic, no. positive (%) | pSS(n=5) | HC(n=5) |
| --- | --- | --- |
| Age (years) ^#^ | 48.4(15) | 33(8) |
| Sex, Female | 5(100) | 5(100) |
| Disease duration, years* | 2.5(5.9) | NA |
| Labial salivary gland with focal lymphocytic sialadenitis and focus score of ≥1 foci/4 mm^2^ | 4(80) | NA |
| Ocualr staining scroe≥5 | 2(40) | NA |
| Schirmer’s test ≤5 mm/5 min in at least 1 eye | 5(100) | NA |
| Unstimulated whole salivary flow rate≤0.1 ml/15 min | 1(20) | NA |
| Anti-SSA/Ro positive | 4(80) | 0(0) |
| Articular | 3(60) | 0(0) |
| Rash involvement | 2(40) | 0(0) |
| Muscular involvement | 0(0) | 0(0) |
| Haematological | 3(60) | 0(0) |
| lymphadenopathy | 1(20) | 0(0) |
| nervous involvement | 0(0) | 0(0) |
| Pulmonary involvement | 1(20) | 0(0) |
| Laboratory characteristic |  |  |
| RF(IU/ml) * | 10.6(75) | NA |
| IgG(g/l) ^#^ | 16.7(3.5) | NA |
| IgM(g/l) ^#^ | 1.2(0.8) | NA |
| IgA(g/l) ^#^ | 2 (1.6) | NA |
| Complement C3(mg/dl) ^#^ | 1.1(0.2) | NA |
| Complement C4(mg/dl) ^#^ | 0.2(0.1) | NA |
| ESR(mm/hr) ^#^ | 26.2(14.6) | NA |
| CRP(mg/l) ^#^ | 2.5(2.6) | NA |
| ESSDAI^#^ | 3(2.4) | NA |

*:median (range);^#^:mean(standard deviation); NA: not applicable; RF: Rheumatoid factor; ESR：Erythrocyte sedimentation rate; CRP: C-reactive protein.

**Supplementary Table-2. Clinical and laboratory features of patients with pSS studied for flow cytometry**

| Clinical characteristic, no. positive (%) | pSS (n=10) | HC (n=10) |
| --- | --- | --- |
| Age (years) ^#^ | 48.8 (10.6) | 40.5 (11.4) |
| Sex, Female | 10 (100) | 10 (100) |
| Disease duration, years* | 3.6 (15.7) | NA |
| Labial salivary gland with focal lymphocytic sialadenitis and focus score of ≥1 foci/4 mm^2^ | 6 (60) | NA |
| Ocualr staining score ≥ 5 | 5 (50) | NA |
| Schirmer’s test ≤ 5 mm/5 min in at least 1 eye | 6 (60) | NA |
| Unstimulated whole salivary flow rate ≤ 0.1 ml/15 min | 4 (40) | NA |
| Anti-SSA/Ro positive | 10 (100) | 0 (0) |
| Articular | 2 (20) | 0 (0) |
| Rash | 2(20) | 0 (0) |
| Muscular involvement | 0 (0) | 0 (0) |
| Haematological | 4 (40) | 0 (0) |
| lymphadenopathy | 1(20) | 0 (0) |
| nervous involvement | 0 (0) | 0 (0) |
| Pulmonary involvement | 2 (20) | 0 (0) |
| Laboratory characteristic |  |  |
| RF(IU/ml) ^#^ | 22 (33) | NA |
| IgG(g/l) ^#^ | 18.1(4.4) | NA |
| IgM(g/l) ^#^ | 1.4 (0.7) | NA |
| IgA(g/l) ^#^ | 2.6 (1.5) | NA |
| Complement C3(mg/dl) ^#^ | 1(0.2) | NA |
| Complement C4(mg/dl) ^#^ | 0.2 (0.1) | NA |
| ESR(mm/hr) ^#^ | 22.3 (21) | NA |
| CRP(mg/l) ^#^ | 0.6 (52) | NA |
| ESSDAI^#^ | 3.8 (2.1) | NA |

*:median (range); ^#^:mean(standard deviation); NA: not applicable; RF: Rheumatoid factor; ESR：Erythrocyte sedimentation rate; CRP: C-reactive protein.

**Supplementary Table-3. Clinical and laboratory features of patients with pSS studied for RT-qPCR**

| Clinical characteristic, no. positive (%) | pSS (n=6) | HC (n=4) |
| --- | --- | --- |
| Age (years) ^#^ | 40.7(10.2) | 39.8 (4.8) |
| Sex, Female | 10(100) | 10(100) |
| Disease duration, years* | 4.8(9.4) | NA |
| Labial salivary gland with focal lymphocytic sialadenitis and focus score of ≥1 foci/4 mm^2^ | 4(67) | NA |
| Ocualr staining score ≥5 | 5(83) | NA |
| Schirmer’s test ≤5 mm/5 min in at least 1 eye | 3(50) | NA |
| Unstimulated whole salivary flow rate≤ 0.1 ml/15 min | 4(7) | NA |
| Anti-SSA/Ro positive | 6(100) | 0(0) |
| Articular | 3(50) | 0(0) |
| Rash | 1(17) | 0(0) |
| Muscular involvement | 0(0) | 0(0) |
| Haematological | 4(67) | 0(0) |
| lymphadenopathy | 2(33) | 0(0) |
| nervous involvement | 0(0) | 0(0) |
| Pulmonary involvement | 2(33) | 0(0) |
| Laboratory characteristic |  |  |
| RF(IU/ml) ^#^ | 32(36) | NA |
| IgG(g/l) ^#^ | 15.1(7.7) | NA |
| IgM(g/l) ^#^ | 1.4(0.4) | NA |
| IgA(g/l) ^#^ | 2.6(1.4) | NA |
| Complement C3(mg/dl) ^#^ | 0.8(0.1) | NA |
| Complement C4(mg/dl) ^#^ | 0.1(0.04) | NA |
| ESR(mm/hr) ^#^ | 17.5(16.3) | NA |
| CRP(mg/l) ^#^ | 0.5(0.3) | NA |
| ESSDAI^#^ | 6.2(2.1) | NA |

*:median (range);^#^:mean(standard deviation);NA: not applicable; RF: Rheumatoid factor; ESR：Erythrocyte sedimentation rate; CRP: C-reactive protein.
